# Transbronchial lung biopsy for the diagnosis of lymphangioleiomyomatosis: the severity of cystic lung destruction assessed by the modified Goddard scoring system as a predictor for establishing the diagnosis

**DOI:** 10.1186/s13023-020-01409-5

**Published:** 2020-05-26

**Authors:** Shouichi Okamoto, Kazuhiro Suzuki, Takuo Hayashi, Keiko Muraki, Tetsutaro Nagaoka, Koichi Nishino, Yasuhito Sekimoto, Shinichi Sasaki, Kazuhisa Takahashi, Kuniaki Seyama

**Affiliations:** 1 Division of Respiratory Medicine, Juntendo University Faculty of Medicine and Graduate School of Medicine, 3-1-3 Hongo, Bunkyo-ku, Tokyo, Japan; 2 The Study Group for Pneumothorax and Cystic Lung Diseases, 4-8-1 Seta, Setagaya-ku, Tokyo, Japan; 3 Division of Radiology, Juntendo University Faculty of Medicine and Graduate School of Medicine, 3-1-3 Hongo, Bunkyo-ku, Tokyo, Japan; 4 Division of Human Pathology, Juntendo University Faculty of Medicine and Graduate School of Medicine, 3-1-3 Hongo, Bunkyo-ku, Tokyo, Japan; 5 Division of Respiratory Medicine, Juntendo Urayasu Hospital, 2-1-1 Tomioka Urayasu-shi, Chiba, Japan

**Keywords:** Bronchoscopy, Lymphangioleiomyomatosis, Modified Goddard scoring system, Transbronchial lung biopsy

## Abstract

**Background:**

A guide of patient selection for establishing the diagnosis of lymphangioleiomyomatosis (LAM) by transbronchial lung biopsy (TBLB) has not been established, although the pathological confirmation of LAM by lung biopsy is desirable, particularly when patients have no additional test results except typical findings of computed tomography (CT) of the chest.

**Methods:**

We retrospectively reviewed the medical records of LAM patients who visited at our hospital from January 2010 to September 2018. We found 19 patients who underwent TBLB and collected the following data to investigate which parameters could predict the TBLB diagnostic positivity for LAM: age, degree of exertional dyspnea, pulmonary function test, cystic lung destruction visually assessed by the modified Goddard scoring system (MGS), serum level of vascular endothelial growth factor-D, and TBLB-related data.

**Results:**

The diagnosis of LAM was established by TBLB in 15 of 19 patients (78.9%) and no serious complications occurred. MGS was significantly higher in the TBLB-positive group than the TBLB-negative group. In LAM patients without pulmonary lymphatic congestion on CT (N = 16), multivariable logistic regression analysis revealed that MGS and FEV_1_/FVC were independent contributing parameters for TBLB diagnostic positivity. However, the analysis of Bayesian inference demonstrated that MGS is a better predictor than FEV_1_/FVC; the probability of establishing diagnosis exceeds 80% if MGS is > 2 (i.e., area of cystic destruction occupies > 25% of lung parenchyma on CT).

**Conclusions:**

MGS may be a helpful and convenient tool to select candidates for TBLB to establish the diagnosis of LAM pathologically.

## Introduction

Lymphangioleiomyomatosis (LAM) is a systemic neoplastic disease that is associated with diffuse cystic lung destruction caused by proliferation and infiltration of smooth muscle-like cells (i.e., LAM cells) [1–3]. LAM mostly affects women of childbearing age and occurs both in a non-heritable form and with tuberous sclerosis complex (TSC) [4]. Dyspnea on exertion, cough, hemoptysis, and chylothorax develop with progression of the disease and recurrent pneumothoraces are common [5, 6].

The characteristic findings of computed tomography (CT) of the chest in LAM are multiple, round and thin-walled cysts presenting with diffuse distribution in the bilateral lungs. This CT appearance is pathognomonic and essential for the diagnosis of LAM [7]. However, pathological confirmation of LAM by lung biopsy is desirable to establish the diagnosis, particularly when patients have no additional diagnostic clues besides typical CT appearance. Furthermore, pathological confirmation of LAM by lung biopsy is recommended before pharmacologic therapy with sirolimus begins in patients for whom the disease was established only by pathognomonic CT appearance.

In an official American Thoracic Society (ATS) and the Japanese Respiratory Society (JRS) clinical practice guideline, transbronchial lung biopsy (TBLB) is conditionally recommended as a less invasive method than surgical lung biopsy when the histopathological diagnosis of LAM is needed [8]. However, appropriate guidelines of patient selection for TBLB have not been determined because of the rarity of this disease. From a pathological point of view, LAM cell nests are usually detected along the walls of lung cysts, in visceral pleura, and in peribronchovascular lesions in the lungs. The increase of cyst burden on CT appearance is well known to correlate with the disease severity that is represented by the deterioration of pulmonary function variables such as forced expiratory volume in 1 s (FEV_1_) and diffusing capacity for carbon monoxide (DLco) [9, 10]. Accordingly, the profusion of cysts on CT appearance reasonably implicates parenchymal LAM burden and therefore it is expected to result in high diagnostic yield when TBLB is performed.

We hypothesized that the modified Goddard scoring system, which uses visual evaluation to assess the degree of cystic lung destruction, could be instrumental to predict the probability of successfully establishing the diagnosis of LAM by TBLB. In this study, we retrospectively reviewed medical records of LAM patients who underwent TBLB to establish the diagnosis of LAM pathologically, and then analyzed the correlation between the diagnostic yield and the modified Goddard scores.

## Methods

From January 2010 to September 2018, 355 patients with LAM who visited at Juntendo University Hospital. All patients were diagnosed according to the diagnostic criteria of the Japanese Respiratory Society [11]. Of these patients, we retrospectively evaluated the medical records of 19 LAM patients who underwent TBLB, chest CT, and pulmonary function tests in our hospital. The following data were extracted: gender, age, the score of the modified Medical Research Council (mMRC) dyspnea scale, smoking history, serum level of vascular endothelial growth factor-D (VEGF-D) before TBLB (measured by an enzyme-linked immunosorbent assay [R&D Systems, Minneapolis, MN, USA]), the presence of extrapulmonary LAM, results of pulmonary function tests, chest CT images, and TBLB-related data (i.e., the number of biopsied specimens, and the associated complications).

Bronchoscopy was performed by pulmonologists using a flexible bronchoscope (Olympus BF 1 T260, Olympus Corporation, Shinjuku-ku, Tokyo, Japan) following local anesthesia with 2% lidocaine. TBLB was conducted from the lower, to middle, to upper lobes in order, with the use of biopsy forceps; at least 2 samples were obtained from each lobe if applicable. TBLB was performed under fluoroscopic guidance.

Biopsied specimens were evaluated by pathologists who had experience with LAM. The histopathological diagnosis of LAM was confirmed based on the presence of foci of LAM cells by hematoxylin and eosin staining, as well as immunohistochemical reactivity with melanoma-associated antigen gp100 (stained by monoclonal antibody clone HMB45), α-smooth muscle actin, and either estrogen or progesterone receptors.

### Evaluation of the severity of cystic lung destruction using the modified Goddard scoring system

Chest CT scans were performed in the supine position with the breath held at full inspiration. Although all examinations of chest CT were performed before TBLB, the CT images of 2 patients had been preserved as X-ray films whereas only digital data were used for the remaining 17 patients. Analyses of chest CT scans in these 2 patients were conducted with digital CT images 3 years after TBLB. The thickness of CT slices in 15 patients was 2 mm, considered high-resolution CT (HRCT), and 5 mm in the remaining 4 patients.

The degree of cystic lung destruction was visually assessed independently by 2 pulmonologists (S.O. and K.S.) and 1 radiologist (K.S.) according to the modified Goddard scoring system [12]. Briefly, 6 images from 3 slice levels (upper, middle, lower in bilateral lung fields) per patient were analyzed and the degree of cystic destruction was scored as follows: normal (score = 0), ≤ 5% affected (score = 0.5), ≤ 25% affected (score = 1), ≤ 50% affected (score = 2), ≤ 75% affected (score = 3), and > 75% affected (score = 4). An average score of all 6 images was calculated as the modified Goddard score (MGS) for each patient. Kendall’s coefficient of concordance for MGS assessed by 3 observers was 0.838 (*P* < 0.001), indicating high inter-observer reliability.

### Statistical analysis

All statistical analyses were performed using R, version 3.4.1. Categorical variables were compared using Fisher’s exact test. The Mann-Whitney test was used to compare non-parametric variables. Student’s t-test was used to compare parametric variables. The correlations between MGS and pulmonary function were examined by Spearman rank correlation test. A multivariable logistic regression was conducted to predict the establishment of diagnosis by TBLB, including MGS and clinical parameters in the model. A *P* value of less than 0.05 was considered significant.

To deduce the probability of the TBLB diagnostic positivity assumed by data on MGS or FEV_1_/ *forced vital capacity* (FVC), we performed Bayesian inference using Stan via the rstan (Version 2.18.2) interface, compiled on C++. Stan uses the No-U-Turn Sampler which extends a type of Markov Chain Monte Carlo (MCMC) algorithm known as Hamiltonian Monte Carlo (HMC) sampling [13]. We used a MCMC chain with 10,000 iterations using the first 1000 as burn-in, which were pooled to represent the posterior distributions of parameter values. A Bernoulli distribution was assumed for the posterior probability distribution and a non-informative uniform distribution for the prior distribution.

## Results

### Patient characteristics

Patient characteristics of all 19 LAM patients are shown in Table 1. The diagnosis of LAM was established by TBLB in 15 of 19 patients (78.9%); thus, the foci of LAM cells was successfully demonstrated in 15 patients (TBLB-positive group) but not in the remaining 4 patients (TBLB-negative group). All patients were female sporadic LAM cases and none of them had a mMRC dyspnea scale score ≥ 3. Five TBLB-positive patients were ex-smokers; each pack-years was 1.4, 3.5, 6, 7 and 34, respectively.

**Table 1:** Characteristics of the study sample

	Total (N = 19)	TBLB		*P* value
		positive (N = 15)	negative (N = 4)	
Age	41 (29–58)	42 (29–58)	40 (37–49)	0.928
mMRC score 0/1/2	2/12/5	2/10/3	0/2/2	0.219
Smoking history ^†^				0.530
never ex-smoker	14 5	10 5 ^†^	4 0	
Serum VEGF-D (pg/mL)	2324.7 (457.3–5932.1)	3138.8 (516.9–5932.1)	2213.6 (457.3–2324.7)	0.263
Extrapulmonary LAM lesions ^‡^	11	8	3	0.603
Pulmonary function tests				
VC (%pred)	101.7 (77.3–126.4)	101.7 (77.3–126.4)	96.8 (79.8–105.4)	0.460
FEV_1_(%pred)	64.8 (37.6–89.0)	62.0 (37.6–89.0)	66.7 (59.3–81.1)	0.539
FEV_1_/FVC (%)	73.4 (35.4–103.4)	71.1 (35.4–103.4)	81.8 (70.0–90.3)	0.307
DLco (%pred)	36.3 (20.9–65.9)	36.3 (26.6–64.4)	38.5 (20.9–65.9)	0.980
MGS	2.72 (0.92–3.61)	2.72 (1.17–3.61)	2.08 (0.92–2.89)	0.017

Values are presented as counts, median values (range)

^†^ Each pack-years is 1.4, 3.5, 6, 7 and 34

^‡^ Extrapulmonary LAM includes lymphangioleiomyoma of the mediastinum, retroperitoneum, and pelvis

Abbreviations: DLco, diffusing capacity of carbon monoxide; FEV_1_, forced expiratory volume in 1 s; FVC, *forced vital capacity*; LAM, lymphangioleiomyomatosis; MGS, modified Goddard score; mMRC, *modified Medical Research Council;* TBLB, transbronchial lung biopsy; VC, vital capacity; VEGF-D, vascular endothelial growth factor-D; %pred, % of the predicted volume

**Fig. 1:**
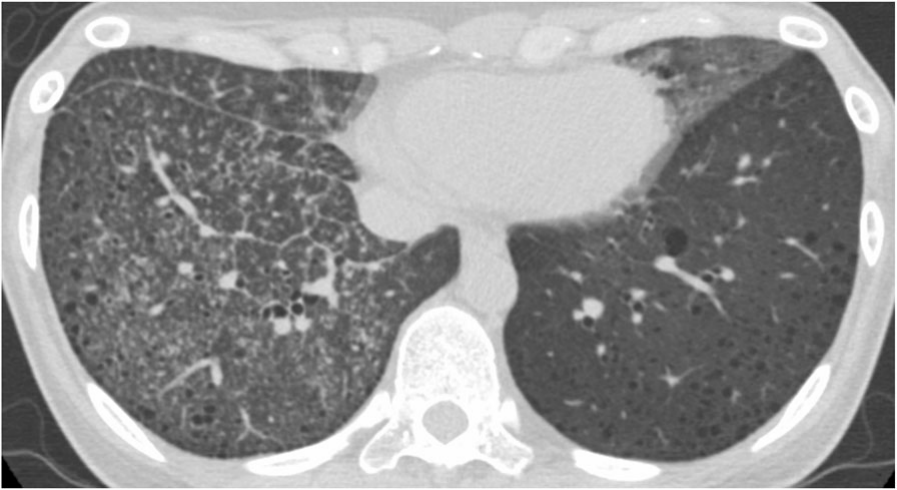
The representative image of a LAM patient with pulmonary lymphatic congestion. Computed tomography of the chest showing small nodules, irregularly increased parenchymal densities, thickening of interlobular septa, a major fissure and peribronchovascular interstitium in the right lower lung, and increased parenchymal densities in the lingula of the left lung. We obtained 6 biopsied specimens from the right lung by TBLB and established the diagnosis of LAM. The MGS of this patient were 0.833 and 1.17, respectively. Abbreviations: LAM, lymphangioleiomyomatosis; MGS, modified Goddard score; TBLB, transbronchial lung biopsy

The chest CT images of all patients demonstrated characteristic CT features of LAM. Two TBLB-positive and 1 TBLB-negative patient showed lymphatic congestion as illustrated in Fig. 1. The serum VEGF-D level was not significantly different between TBLB-positive and -negative groups.

**Fig. 2:**
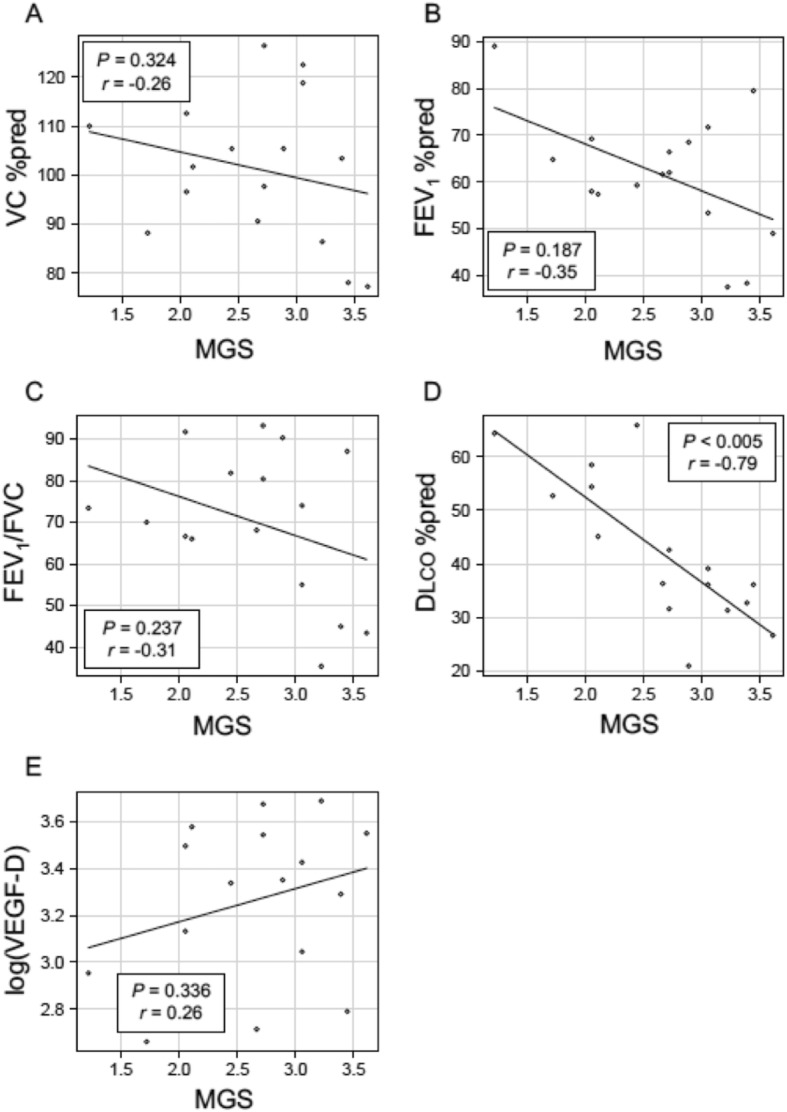
Relationships between MGS and clinical parameters in 16 LAM patients without pulmonary lymphatic congestion. A, VC %pred; B, FEV_1_%pred; C, FEV_1_/FVC; D, DLco %pred; and E, log (VEGF-D). MGS was significantly negatively correlated with DLco %pred (D). Abbreviations: DLco, diffusing capacity of carbon monoxide; FEV_1_, forced expiratory volume in 1 s; FVC, *forced vital capacity*; LAM, lymphangioleiomyomatosis; log (VEGF-D), logarithmic value of serum vascular endothelial growth factor-D (pg/ml); MGS, modified Goddard score; VC, vital capacity; %pred, percentage of the predicted value

We found that MGS was significantly higher in the TBLB-positive group than the TBLB-negative group (Table 1). For the full study sample (N = 19), MGS was significantly negatively correlated with FEV_1_% of the predicted volume (%pred) (*P* = 0.034, *r* = − 0.49) and DLco %pred (*P* = 0.022, *r* = − 0.52), but was not significantly related to either vital capacity (VC) %pred or log (VEGF-D) (Fig. S1, Supplementary Information)). Since MGS is a visual assessment of cystic destruction, we excluded 3 LAM patients who had pulmonary lymphatic congestion from analyzing the association between TBLB results and various clinical parameters including MGS. In the remaining group of 16 LAM patients, we found that MGS was significantly negatively correlated only with DLco %pred (*P* < 0.005, *r* = − 0.79) (Fig. 2).

### Diagnosis of LAM by TBLB

The TBLB procedure was performed from the right lung in 17 patients and the left lung in 2 patients. A total of 92 tissue specimens were biopsied from the 19 patients during the TBLB procedures. No significant difference was found between TBLB-positive and -negative groups; the median number of biopsied specimens was 5 (range 3–6) in the positive group vs. 5.5 (range 4–6) in the negative group (*P* = 0.427).

No other serious events such as pneumothorax or pneumonia occurred in the full study sample. Two patients had transient fevers within 1 day after bronchoscopy. Two episodes of bleeding with moderate severity occurred after biopsy but both were managed with topical endobronchial application of epinephrine.

To identify potential clinical parameters predicting the TBLB diagnostic positivity for LAM, we performed a multivariable logistic regression analysis, incorporating DLco %pred, FEV_1_/FVC, FEV_1_%pred, log (VEGF-D), and MGS in the model. In LAM patients without pulmonary lymphatic congestion on chest CT (N = 16), the TBLB diagnostic positivity for LAM was significantly influenced by MGS (*P* = 0.018) and FEV_1_/FVC (*P* = 0.006) independently (data not shown).

**Fig. 3:**
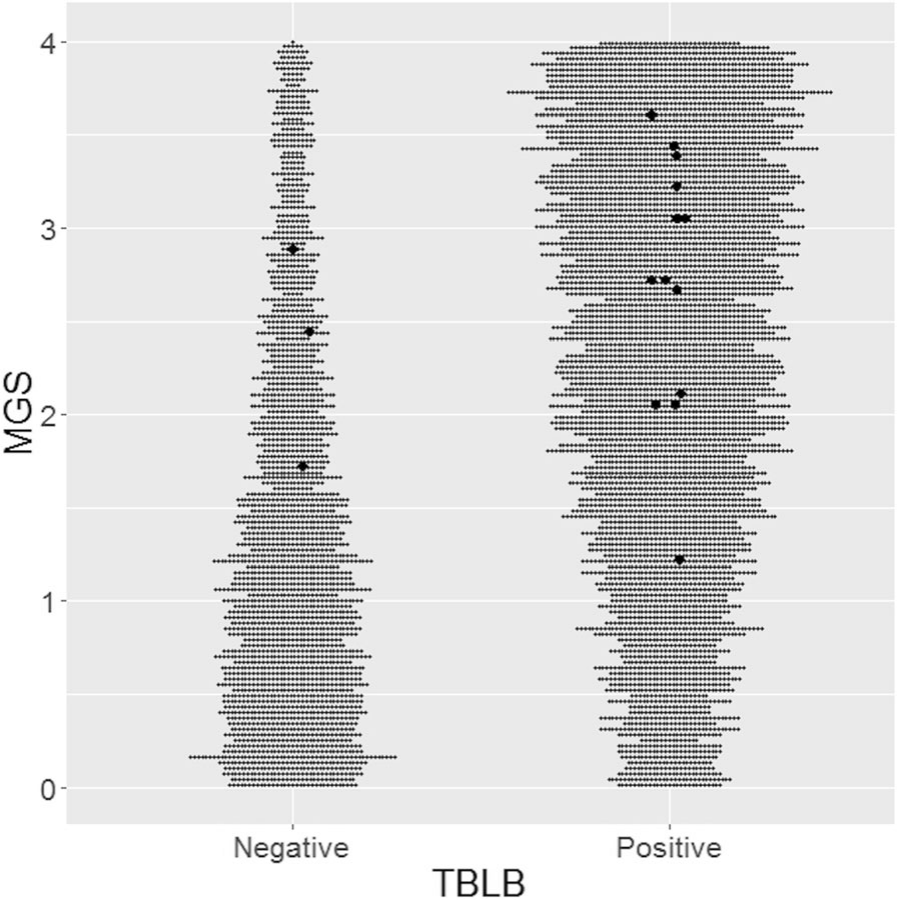
The distribution of MGS in TBLB-positive vs. TBLB-negative patients by HMC sampling. Small gray dots show the posterior probability distributions generated by HMC sampling. Large black dots indicate the data derived from 16 LAM patients without pulmonary lymphatic congestion. The width of TBLB-positive gray dots proportionally increased as the MGS increased whereas TBLB-negative gray dots showed the opposite relationship with MGS. Note that TBLB-positive patients primarily had MGS > 2. Abbreviations: HMC, Hamiltonian Monte Carlo; LAM, lymphangioleiomyomatosis; MGS, modified Goddard score; TBLB, transbronchial lung biopsy

Next, to validate which parameter, MGS or FEV_1_/FVC, is more suitable to predict TBLB diagnostic positivity for LAM, we estimated the probability of diagnostic positivity generated by HMC sampling based on data from the LAM patients without lymphatic congestion on chest CT (N = 16). As shown in Table 2, the probability of TBLB diagnostic positivity was estimated to increase when MGS increased. Similarly, the distribution of sampling plots showed that the direction of increase or decrease of TBLB diagnostic positive spots was identical to that of MGS (Fig. 3). In concordance with the estimation that the probability of TBLB positivity was more than 0.832 in the levels of MGS > 2 (Table 2), almost all TBLB-positive patients had MGS > 2 (Table 1). FEV_1_/FVC was also able to estimate the TBLB-diagnostic positivity for LAM (Table S1, Supplementary Information). However, the distribution of sampling plots showed weaker correlations with the change in FEV_1_/FVC as compared with the change in MGS (Fig. S2, Supplementary Information)). Accordingly, we concluded that MGS was a more suitable predictor than FEV_1_/FVC.

**Table 2:** The probability of TBLB diagnostic positivity for LAM by level of MGS

	Probability of diagnostic positivity for LAM	95% credible interval
3 < MGS≦4	0.910	0.897–0.921
2 < MGS≦3	0.832	0.816–0.847
1 < MGS≦2	0.606	0.586–0.626
0 < MGS≦1	0.393	0.373–0.413

The data was generated by Hamiltonian Monte Carlo sampling based on data from 16 LAM patients without lymphatic congestion

95% credible interval shows the 2.5 and 97.5% percentiles of distribution

Abbreviations: LAM, lymphangioleiomyomatosis*;* MGS, modified Goddard score; TBLB, transbronchial lung biopsy

## Discussion

Our retrospective study found that MGS, a score representing visual assessment of cystic destruction in the lungs of LAM patients, may be a reliable predictor for establishing histopathological diagnosis of LAM by TBLB. The probability of TBLB diagnostic positivity was estimated to increase or decrease proportionally with the level of MGS; the probability of establishing histopathological diagnosis exceeded 80% if MGS was > 2 (i.e., area of cystic destruction occupied > 25% of lung parenchyma on CT). In our analysis of the full study sample, MGS was significantly negatively correlated with FEV_1_%pred and DLco %pred, representing the disease severity of LAM [5, 14]. Furthermore, multivariable logistic regression analysis identified both MGS and FEV_1_/FVC as independently contributing parameters to TBLB diagnostic positivity. However, the analysis of Bayesian inference by HMC sampling revealed that MGS is a better predictor for establishing the diagnosis of LAM by TBLB than FEV_1_/FVC.

TBLB is a less invasive method than video-associated thoracoscopic surgery-guided lung biopsy; the complication and the mortality rates are 2.1% vs. 9.6–19.1 and 0.004% vs. 1.5–4.4%, respectively [15–18]. Therefore, the official ATS/JRS clinical practice guideline recommends conducting TBLB prior to a surgical lung biopsy for patients with characteristic of LAM, but none of certain clinical features that are suggestive of LAM. Those clinical features are: the presence of TSC, renal angiomyolipoma(s), elevated serum VEGF-D ≧800 pg/mL, chylous effusion (pleural or ascites) confirmed by tap and biochemical analysis of the fluid, lymphangioleiomyomas, or demonstration of LAM cell clusters or LAM cells on cytological examination of chylous effusions or aspiration of lymph nodes [8]. However, neither the criteria for patient selection for TBLB nor recommendations for the number of tissue specimens that should be biopsied per TBLB procedure have been established.

The diagnostic yield by TBLB in our retrospective study was 78.9%, which is comparable to or higher than the past case series reports [19–23]. Our high diagnostic yield is likely derived from selection bias, as 15 (78.9%) of 19 patients had MGS > 2. In the light of theoretical considerations regarding the TBLB procedure, advanced-stage LAM patients whose burden of LAM cells becomes more extensive would likely result in histopathologic diagnosis. In addition, as the number of tissue specimens biopsied per TBLB procedure increases, the probability of establishing histopathological diagnosis of the disease would increase. However, complications from TBLB are expected to occur more in the LAM patients with severe cystic destruction, as well as the number of biopsy procedures per TBLB increases.

We attempted to find the level of severity of LAM that could balance the benefit and risk of TBLB, and then tried to select LAM patients whose MGS was between 2 and 3 (i.e., corresponding to cystic destruction > 25%, but < 75% of lung parenchyma). Complications that were experienced in this “biased cohort” included transient fever (N = 2, 10.5%), controllable bleeding (N = 2, 10.5%) and no pneumothorax seems to be acceptable because the overall risk of any complication of TBLB for LAM was reported 14% [21] and the risk of pneumothorax with transbronchial biopsy for peripheral lung lesions was estimated to be about 2% [24, 25].

This study had several limitations. First, there are some issues regarding the study population. Our study was conducted retrospectively on a small sample due to the rare occurrence of this disease. Additionally, our cohort study had selection bias so that the distribution of MGS was generally between MGS ≥ 2 and MGS < 4. Furthermore, we excluded 3 LAM patients with pulmonary lymphatic congestion from the statistical analysis, including 2 TBLB-positive patients (MGS 2.89 and 1.17, respectively) and 1 TBLB-negative patient (MGS 0.917), because the significance of this condition in terms of the burden of LAM cells remains uncertain. Second, the MGS is based on a 3-slice assessment which may not reflect the degree of cystic destruction of the entire LAM lungs. However, it is a simple measure that can be obtained in any facility. Third, for 2 patients the MGS was determined using HRCT images obtained after the TBLB was performed because only film-based CT images were available prior to the procedure. However, we believe there is only a small possibility that the degree of cystic destruction was overestimated, as the interval from TBLB to acquisition of CT images was 3 years in both patients. Further studies are needed to clarify the diagnostic positivity by TBLB in LAM patients, especially for those whose cystic destruction is mild (i.e., MGS < 2) or who have pulmonary lymphatic congestion.

## Conclusions

In conclusion, we found that MGS was significantly associated with the definitive diagnosis of LAM by TBLB. Therefore, the MGS may be an effective and convenient tool that will help clinicians select candidates for TBLB to establish histopathological diagnosis of LAM. Patients with MGS between 2 and 3 (i.e., cystic destruction occupying between 25 and 75% of lung parenchyma), have a high probability of establishing a diagnosis with an acceptable risk of complications.

## Supplementary information

**Additional file 1: Fig. S1** Relationships between MGS and clinical parameters in LAM patients (N = 19). **Fig. S2** The distribution of FEV_1_/FVC in TBLB-positive vs. TBLB-negative patients by HMC sampling. **Table S1** The probability of TBLB diagnostic positivity for LAM by level of FEV_1_/FVC (%).

## Data Availability

The datasets used and/or analyzed during the current study are available from the corresponding author on reasonable request.

## Abbreviations

ATS: American Thoracic Society
CT: Computed tomography
DLco: Diffusing capacity for carbon monoxide
FEV_1_: Forced expiratory volume in 1 s
FVC: *Forced vital capacity*
HMC: Hamiltonian Monte Carlo
HRCT: High-resolution computed tomography
JRS: Japanese Respiratory Society
LAM: Lymphangioleiomyomatosis
MCMC: Markov Chain Monte Carlo
MGS: Modified Goddard score
mMRC: Modified Medical Research Council
TBLB: Transbronchial lung biopsy
TSC: Tuberous sclerosis complex
VC: Vital capacity
VEGF-D: Vascular endothelial growth factor-D
