# Supplementary material for: Transbronchial lung biopsy for the diagnosis of lymphangioleiomyomatosis: the severity of cystic lung destruction assessed by the modified Goddard scoring system as a predictor for establishing the diagnosis
Source: Orphanet J Rare Dis. 2020 May 26;15:125. doi: 10.1186/s13023-020-01409-5 (PMC7249378; doi:10.1186/s13023-020-01409-5)
Supplement: Supplementary file 1 — Additional file 1: Fig. S1 Relationships between MGS and clinical parameters in LAM patients (N = 19). Fig. S2 The distribution of FEV1/FVC in TBLB-positive vs. TBLB-negative patients by HMC sampling. Table S1 The probability of TBLB diagnostic positivity for LAM by level of FEV1/FVC (%). [file 13023_2020_1409_MOESM1_ESM.docx]

**Supplementary information**

**Fig. S1**

Relationships between MGS and clinical parameters in LAM patients (N = 19).


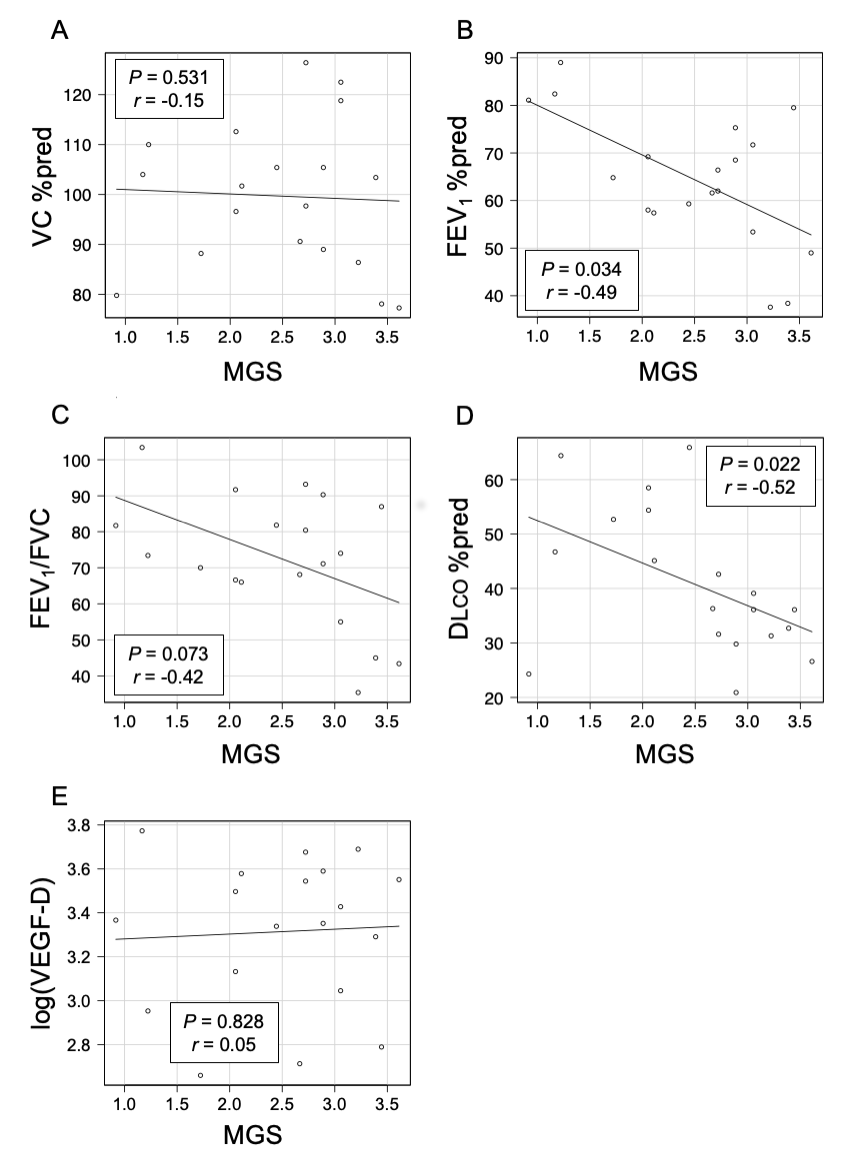


A, VC %pred; B, FEV_1_ %pred; C, FEV_1_/FVC; D, DLco %pred; and E, log(VEGF-D). MGS was significantly negatively correlated with FEV_1_ %pred (B) and DLco %pred (D). There was a trend toward a negative correlation between FEV_1_/FVC (C) and MGS.

Abbreviations: DLco, diffusing capacity of carbon monoxide; FEV_1_, forced expiratory volume in 1 second; FVC, forced vital capacity; LAM, lymphangioleiomyomatosis; log(VEGF-D), logarithmic value of serum vascular endothelial growth factor-D (pg/ml); MGS, modified Goddard score; VC, vital capacity; %pred, percentage of the predicted value.

**Fig. S2**

The distribution of FEV_1_/FVC in TBLB-positive vs. TBLB-negative patients by HMC sampling.


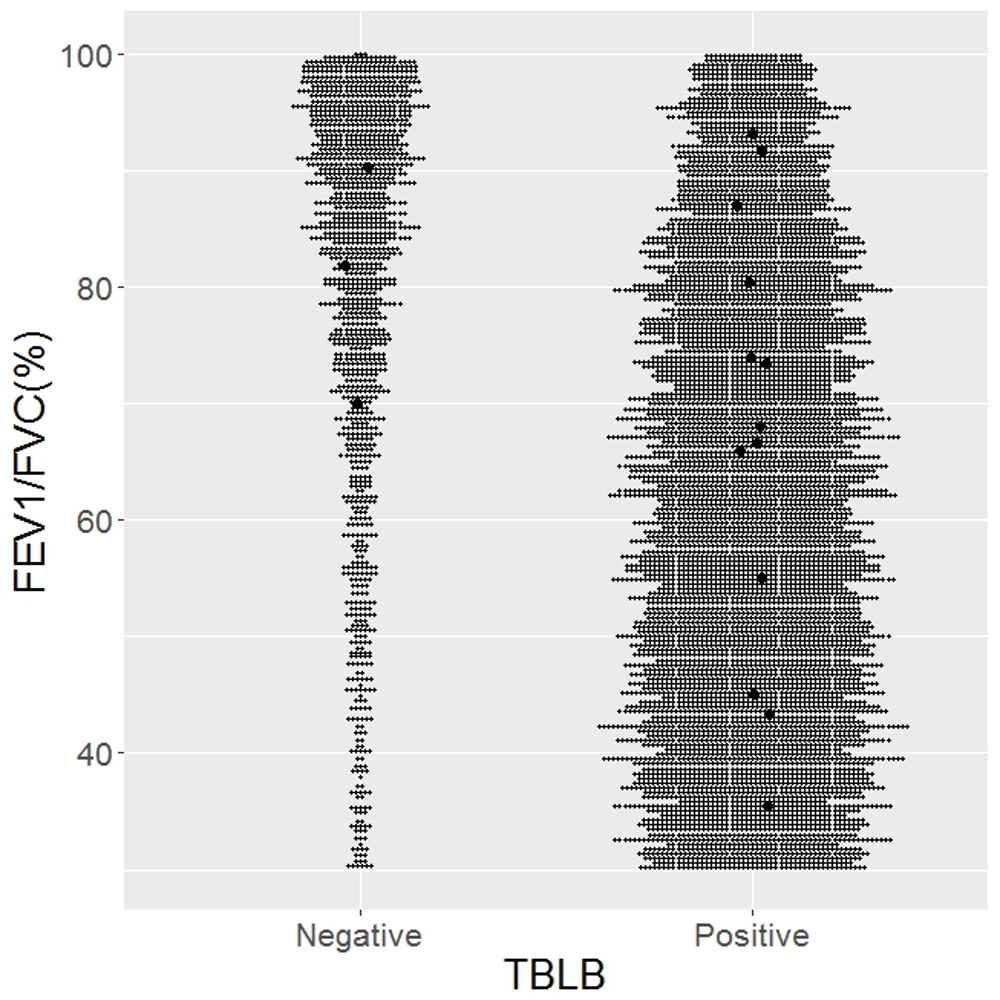


Small gray dots show the posterior probability distribution generated by HMC sampling. Large black dots indicate the data derived from 16 LAM patients without pulmonary lymphatic congestion.

Abbreviations: FEV_1_, forced expiratory volume in 1 second; FVC, forced vital capacity; HMC, Hamiltonian Monte Carlo; LAM, lymphangioleiomyomatosis; TBLB, transbronchial lung biopsy.

**Table S1**

The probability of TBLB diagnostic positivity for LAM by level of FEV_1_/FVC (%).

|  | Probability of diagnostic positivity for LAM | 95% credible interval |
| --- | --- | --- |
| 90≦FEV_1_/FVC<100 | 0.564 | 0.536–0.592 |
| 80≦FEV_1_/FVC<90 | 0.717 | 0.692–0.742 |
| 70≦FEV_1_/FVC<80 | 0.823 | 0.801–0.843 |
| 60≦FEV_1_/FVC<70 | 0.904 | 0.887–0.920 |
| 50≦FEV_1_/FVC<60 | 0.928 | 0.912–0.941 |
| 40≦FEV_1_/FVC<50 | 0.948 | 0.935–0.959 |

The data was generated by Hamiltonian Monte Carlo sampling based on data from 16 LAM patients without lymphatic congestion

95% credible intervals show 2.5% and 97.5% percentiles of distribution.

Abbreviations: FEV_1_, forced expiratory volume in 1 second; FVC, forced vital capacity; LAM, lymphangioleiomyomatosis; TBLB, transbronchial lung biopsy.
